# Potential Application of Optogenetic Stimulation in the Treatment of Pain and Migraine Headache: A Perspective from Animal Studies

**DOI:** 10.3390/brainsci9020026

**Published:** 2019-01-29

**Authors:** Sufang Liu, Yuanyuan Tang, Ying Xing, Phillip Kramer, Larry Bellinger, Feng Tao

**Affiliations:** 1 Department of Biomedical Sciences, Texas A&M University College of Dentistry, Dallas, Texas 75246, USA; sufang.liu@tamhsc.edu (S.L.); yuanyuan8402@163.com (Y.T.); PKramer@tamhsc.edu (P.K.); LBellinger@tamhsc.edu (L.B.); 2 Department of Physiology, Zhengzhou University School of Medicine, Zhengzhou 450001, China; xingyingprofessor@gmail.com; 3 School of Basic Medical Sciences, Xinxiang Medical University, Xinxiang 453003, China; 4 Center for Craniofacial Research and Diagnosis, Texas A&M University College of Dentistry, 3302 Gaston Avenue, Dallas, Texas 75246, USA

**Keywords:** migraine-like pain, optogenetics, neuronal activity modulation, neuronal excitation, neuronal inhibition

## Abstract

Optogenetic manipulation is uniquely useful in unraveling the functional organization of neuronal circuits in the central nervous system by enabling reversible gain- or loss-of-function of discrete populations of neurons within restricted brain regions. This state-of-the-art technology can produce circuit-specific neuromodulation by overexpressing light-sensitive proteins (opsins) in particular cell types of interest. Here, we discuss the principle of optogenetic manipulation and its application in pain research using animal models, and we also discuss how to potentially use optogenetic stimulation in the treatment of migraine headache in the future.

## 1. Introduction

Migraine headache is a common neurovascular disorder [1]. Despite its prevalence, most therapies for migraine headache are either ineffective or inadequate with a number of side effects. Neuromodulatory approaches, such as electrical or magnetic stimulation, have been shown to be effective in producing analgesic-like effects by altering nociceptive transmission in the central nervous system (CNS) [2,3,4], and both approaches have been recently employed for the treatment of migraine headache in humans [5,6,7]. As we know, however, there are different types of neurons in the CNS, and stimulation of these neurons may produce different outcomes. Neuromodulatory approaches using electrical or magnetic stimulation can alter neuronal activity but cannot specifically activate certain type of neurons. Optogenetics has been developed as an advanced neuromodulation tool to specifically control neuronal activity with spatial and temporal precision [8]. By expressing light-sensitive proteins in specific neurons and delivering light with relevant wavelengths, this cutting-edge technique can be used to investigate the neurobiological mechanisms and circuitry underlying nociceptive transmission, which can then be targeted for the development of new therapies for chronic pain conditions in humans. In this review, we will discuss the principle of optogenetic manipulation in context of preclinical models of pain and its potential application for the treatment of migraine headache.

## 2. The Principle of Optogenetic Manipulation

Optogenetic manipulation can produce gain- or loss-of-function in specific type of cells following light application [8]. The main components of an optogenetic tool include a light-sensitive protein (opsin) and a vector to specifically express the opsin on certain type of neurons in a site of interest [8]. Opsins are divided into two groups: ion channels and G-protein-coupled receptors. The most commonly used light-sensitive protein is channelrhodopsin [9]. The first opsin used for optogenetic excitation, channelrhodopsin-2 (ChR2), is a transmembrane protein that causes transmembrane cation channels to open [10]. Upon stimulating by blue light (472 nm), it produces membrane depolarization and neuronal excitation [8,11]. The cation conductance is primarily dependent on fast opening and closing of the channels, which limits the time frame of neuronal activation [12]. Thus, short timescales are the main kinetic limitation of optogenetic manipulation. Some mutations have been made to optimize channel kinetics. For instance, mutations of residue glutamate 123 to threonine or alanine in ChR2 have been made for optogenetic stimulation with more than 40 Hz [8]. On the other hand, optogenetic inhibition of neuronal activity can be produced by stimulation of yellow light (590 nm) on the light-sensitive protein halorhodopsins (e.g., NpHRs), a chloride pump, which leads to hyperpolarization of the neuron [13,14]. Stimulating NpHRs decreases the spiking rate of neurons [15]. When NpHRs are stimulated by yellow light, the neuron expressing NpHRs hyperpolarizes because of chloride ions flowing into the neuron, which inhibits neuronal activity. Currently, three generations of NpHRs have been used. The third-generation NpHR (eNpHR3.0) is now the most commonly used because it is the most stable with longer time scales [14]. 

Viral vectors have been shown to be very powerful in mediating widespread gene expression in the brain [15]. Both lentiviral vectors and adeno-associated viral (AAV) vectors have been extensively applied in studies employing optogenetic manipulation. By using viral vectors, we can specifically express light-sensitive proteins in certain type of neurons with long-term stability and at relatively high levels. Lentiviral and AAV vectors have different features: (1) Lentiviral vectors are permanently integrated into the genome of host neurons, while AAV vectors are mostly expressed as an extra-chromosome in the host neurons; (2) The inserted construct lengths are different: it is up to 5 kb for AAV vectors, whereas it can be as large as 10 kb for lentiviral vectors [16]; (3) Compared with lentiviral vectors, AAV vectors are more stable at the insertion site and thus are less likely to mutate [8,17]. Lentiviral vectors may increase the efficiency of gene delivery in the brain by enhancing trafficking [18]. Importantly, viral transduction of optogenetic constructs does not require the assistance of exogenous chemical cofactors that could manipulate cellular behaviors in vivo [19]. 

**Figure 1:**
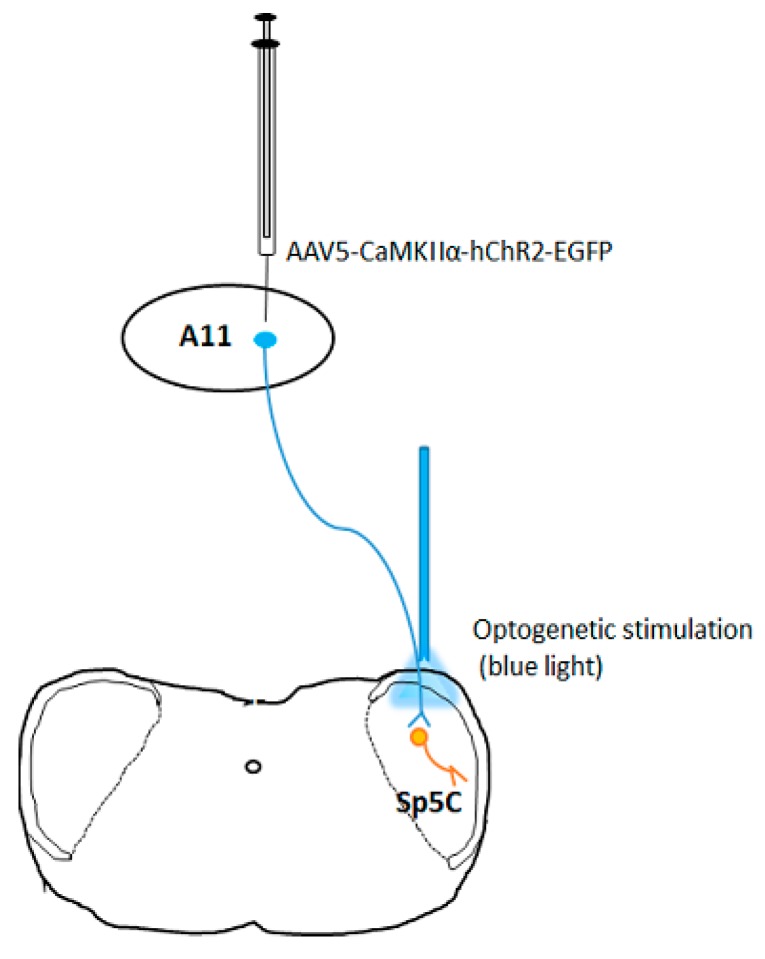
Optogenetic excitation of neuronal activity in the A11-Sp5C pathway.

Optogenetic manipulation can produce circuit-specific neuromodulation by overexpressing light-sensitive proteins (opsins) in particular cell types of interest. This is accomplished either by the use of viral vectors that infect only certain types of neurons through cell type-specific promoters, such as calcium/calmodulin-dependent protein kinase IIα (CaMKIIα), which will localize opsins to excitatory neurons [20], or by targeted use of viral vectors that express their transgenes in a Cre-dependent manner (Cardin et al., 2010). For instance, we can inject an AAV vector with CaMKIIα promoter (AAV5-CaMKIIα-hChR2-EGFP) to specifically express ChR2 on excitatory neurons in the hypothalamic A11 nucleus and then optogenetically stimulate (with blue light) axon terminals of excitatory neurons in the spinal trigeminal nucleus caudalis (Sp5C, see Figure 1, unpublished), thereby producing specific modulation of neuronal activity in the A11−Sp5C pathway. Because depolarizing opsin ChR2, a light-gated cation channel, allows positively charged ions (primarily Na^+^) to flow into intracellular space to induce neuronal excitation when stimulated by blue light and conversely, the hyperpolarizing opsin eNpHR3.0, a chloride pump activated by yellow light, allows Cl^-^ to flow into neurons to produce neuronal inhibition, it is possible to study the behavioral consequences of activating or inhibiting the same ensembles of neurons by expressing the two light-sensitive proteins on the same neurons [14]. 

Stimulation frequency and duration are critical factors in optogenetic neuromodulation. A previous study reported that while low frequency of optogenetic stimulation increases the release of amino acid neurotransmitters, higher frequency of optogenetic stimulation increases the release of both amino acid neurotransmitters and neuropeptides [21]. Another study showed that optogenetic stimulation of dopaminergic neurons in ventral tegmental area with low (5 Hz) and high (50 Hz) frequencies produces different release patterns [22]. In addition, high frequency optogenetic stimulation may cause tissue damage [23], such as heating-induced brain damage, which will undoubtedly alter neuronal activity in a non-meaningful way. Therefore, choosing the right frequency parameters for optogenetic stimulation is of critical importance. In addition, other stimulation parameters, including duration and current intensity should be optimized before applying optogenetic stimulation given that continuous light stimulation or stimulation at high intensities can cause abnormal neuronal firing patterns and even excitotoxicity. Optogenetic stimulation with optimized parameters will be expected to yield the maximal results without adverse effects.

## 3. The Applications of Optogenetic Manipulation

### 3.1. Application in Pain Research

Optogenetic manipulation can be used to specifically regulate neuronal activity in neural circuits [24]. Combined with a conditional transgenic approach that expresses opsins in a specific neuronal circuit, optogenetic manipulation has been extensively used to delineate connectivity of neuronal ensembles and their signaling pathways in the CNS. Recently, optogenetic manipulation has provided new insights into the neurocircuitry mechanisms underlying nociceptive transmission. Crock et al. reported that optogenetic activation of metabotropic glutamate receptor 5 in the central nucleus of the amygdala (CeA) markedly increases visceral pain-related responses in a mouse model of distension-induced pain [25]. In addition, by inserting the intracellular domain of a native µ-opioid receptor into the intracellular sequence of channelrhodopsin, an optogenetically excited µ-opioid receptor has been created, which allows for optogenetic manipulation in preclinical models aimed toward the development of novel treatment approaches for chronic pain in humans [26]. 

Currently there are different types of devices for optogenetic stimulation, including classic and portable devices. The classic device needs to be implanted into stimulation sites, which could cause potential inflammation and nerve injury. The portable microdevice can be used for wireless light stimulation, which enables neuronal activity to be modulated noninvasively. The effect of optogenetic stimulation usually lasts for a short time period, thus repeated optical stimulation is likely necessary to produce analgesic-like effects. Optogenetic stimulation can be used to modulate nociceptive signaling remotely [27,28]. For example, optovin, a light-sensitive ligand for TRPA1 (a member of transient receptor potential (TRP) family), can alter the function of nociceptive neurons in response to direct light stimulation [29]. Upon photo-activation by illumination, optovin can form reversible bonds with TRPA1, which will cause cation influx into neurons and alter membrane potentials [29]. Moreover, optovin activates TRPA1 following light stimulation in cultured HEK cells expressing human TRPA1 [27]. Quaternary ammonium-azobenzene-quaternary ammonium (QAQ), another small photoisomerizable molecule, is also sensitive to light stimulation [28]. By blocking cation channels intracellularly, light stimulation of QAQ can inhibit the induction of action potentials in nociceptive neurons. Thus, we are able to use QAQ to decrease the firing of nociceptive neurons, thereby inhibiting nociceptive transmission [28]. With the discoveries of optovin and QAQ, we may develop effective optopharmacological therapies for clinical use [27,28,29]. On the other hand, optopharmacological regulation of ion channel gating can be used to investigate the function of different ion channel subunits [30,31].

Recently, optogenetic manipulation has also been used to modulate nociceptive signaling in freely behaving mice [32,33]. Selective activation of sodium channel Nav1.8(+) afferents in vivo by optogenetics induces central sensitization and conditioned place aversion, which provides a novel approach to investigate plasticity in nociceptive circuits [32]. In this seminal study, long-term potentiation was similarly evoked by light activation of the same afferents in isolated spinal cord preparations [32], demonstrating the potential for long-term clinical use. In another study by Iyer and colleagues, an optogenetic strategy has been used to bidirectionally control nociceptors in freely moving mice. Intra-sciatic nerve injection of AAV-linked excitatory opsin produced light-inducible acute pain-like behaviors as well as place aversion. In contrast, viral delivery of an inhibitory opsin produced light-inducible inhibition of the acute pain-like behaviors and reversed mechanical allodynia and thermal hyperalgesia in a model of neuropathic pain [33]. These results demonstrate the optical control of nociception and central sensitization in behaving animals and suggest that specific classes of afferents contribute to central sensitization in nociceptive transmission. Therefore, optogenetic manipulation is a powerful approach for understanding the central mechanisms underlying nociceptive transmission in preclinical animal models and translating these findings into treatments for chronic pain conditions in humans.

### 3.2. Potential Application in the Treatment of Migraine Headache

Although electrical and magnetic neuromodulation techniques produce inhibitory effects on migraine headache, non-specific neuronal activation could compromise their effectiveness and may induce unnecessary side effects. Optogenetic manipulation can specifically modulate neuronal activity with spatial and temporal precision, thereby providing new insights into neural circuitry mechanisms underlying the analgesic effects produced by electrical and magnetic stimulation in the CNS. Furthermore, through either hyperpolarizing opsin-mediated neuronal inhibition of trigeminovascular sensory signaling or depolarizing opsin-mediated neuronal activation of endogenous analgesic system, optogenetic manipulation might be developed as a more efficient tool to treat migraine headache. 

The medial prefrontal cortex (mPFC) has been implicated in top-down control of sensory signaling and affective pain processing. Lee et al. reported that optogenetic activation of the mPFC produces strong anti-nociceptive effects in a rat model of persistent neuropathic pain [34]; that study further provides evidence to show that the projection from the mPFC to the nucleus accumbens is involved in the anti-nociceptive effects induced by optogenetic activation of the neuronal circuit [34]. Another study observed enhanced feedforward inhibition in the prelimbic mPFC in a neuropathic pain model and showed that optogenetic inhibition of GABAergic interneurons decreased pain responses in freely moving mice [35], which identifies GABAergic circuitry in the prelimbic mPFC as a potential target for pain treatment in humans. A recent study found that enhanced feedforward inhibition also exists in the infralimbic mPFC using a model of arthritis induction in rats [36]. This study further demonstrated that rescue of impaired endocannabinoid-dependent mGluR5 function in the infralimbic mPFC can restore mPFC output and cognitive-like functions and inhibit pain [36]. In addition, optogenetic activation of excitatory neurons in contralateral prelimbic mPFC exerts analgesic- and anxiolytic-like effects in mice [37]. It was shown that knocking down cyclin-dependent kinase 5 reverses chronic pain-induced inhibition of the intrinsic excitability of excitatory neurons in the prelimbic mPFC and alleviates pain behaviors [37]. Therefore, optogenetic manipulation of neuronal function in the mPFC might be developed into a new neuromodulation approach for the treatment of intractable chronic pain in humans, including migraine headache. Recently, we reported that optogenetic manipulation of dopamine receptors D1 and D2 in the Sp5C produces opposite effects on nerve injury-induced head withdrawal threshold reduction whereas specific optogenetic excitation of dopaminergic neurons in the hypothalamic A11 nucleus attenuates this response via the activation of D2 receptors in the Sp5C [38].

Cortical spreading depression (CSD) has been demonstrated to be an important mechanism by which migraine headache occurs with an aura [39]. Preventing or blocking CSD has become a common mechanism shared by currently available anti-migraine drugs [40,41,42]. Thus, CSD could be a target for developing an effective therapy for migraine headache. Optogenetic technology is useful not only in migraine research, but also in creating a potential approach for the treatment of migraine headache. Optogenetics has been used to trigger CSD events in freely moving animals to mimic migraine-relevant neuronal silencing in the cerebral cortex for mechanistic studies. The method involves using transgenic mice in which ChR2 is expressed widely in the brain including cerebral cortex under control of neuronal Thy1 promoter [43] combined with optical stimulation through the intact skull [44]. Meanwhile, multiunit activities recorded by a multi-channel system can be employed to confirm the induction of CSD events in the brain. Using this model, a novel optogenetic manipulation-based therapy for migraine headache could be developed by specifically regulating CSD with optical stimulation to prevent or block CSD.

## 4. Conclusions

Optogenetic stimulation is a powerful tool in unraveling neuronal circuits in the CNS. Combining this revolutionary technology with electrophysiological recording will enable us to investigate the central mechanisms underlying pain processing in animal models, which will provide significant insights for novel pain therapy development. Optogenetic manipulation-based circuit-specific neuromodulation approach could be developed into a new neuromodulation approach for the treatment of chronic pain including migraine headache. In the future, the stimulation parameters including frequency, duration and current intensity need to be optimized to obtain the maximal efficacy. Thus, more evaluations will be needed to fine-tune these parameters before we use optogenetic stimulation to treat patients with chronic pain including migraine headache in the clinic.
